# Outcomes of Patients with Malignancy Admitted to the Intensive Care Units: A Prospective Study

**DOI:** 10.1155/2021/4792309

**Published:** 2021-09-01

**Authors:** Hazem I. Assi, Nour Abdul Halim, Ibrahim Alameh, Jessica Khoury, Vicky Nahra, Fares Sukhon, Maya Charafeddine, Clara El Nakib, Nour Moukalled, Maroun Bou Zerdan, Pierre Bou Khalil

**Affiliations:** ^1^Department of Internal Medicine, Division of Hematology and Oncology, Naef K. Basile Cancer Institute, American University of Beirut Medical Center, Beirut, Lebanon; ^2^Department of Internal Medicine, Division of Pulmonary and Critical Care, American University of Beirut Medical Center, Beirut, Lebanon

## Abstract

**Introduction:**

Decisions regarding whether advanced cancer patients should be admitted to the ICU are based on a complex suite of considerations, including short- and long-term prognosis, quality of life, and therapeutic options to treat cancer. We aimed to describe demographic, clinical, and survival data and to identify factors associated with mortality in critically ill advanced cancer patients with nonelective admissions to general ICUs.

**Materials and Methods:**

Critically ill adult (≥18 years old) cancer patients nonelectively admitted to the intensive care units at the American University of Beirut Medical Center between August 1^st^ 2015 and March 1^st^ 2019 were included. Demographic, clinical, and laboratory data were prospectively collected from the first day of ICU admission up to 30 days after discharge. This study was strictly observational, and clinical decisions were left to the discretion of the ICU team and attending physician.

**Results:**

272 patients were enrolled in the study between August 1^st^ 2015 and March 1^st^ 2019, with an ICU mortality rate of 43.4%, with the number rising to 59% within 30 days of ICU discharge. The mean length of stay in our ICU was 14 days (IQR: 1–120) with a median overall survival of 22 days since the date of ICU admission. The major reasons for unplanned ICU admission were sepsis/septic shock (54%) and respiratory failure (33.1%). Cox regression analysis revealed 7 major predictors of poor prognosis. Direct admission from the ED was associated with a higher risk of mortality (48.9%) than being transferred from the floor (32.6%) (*p*=0.014).

**Conclusion:**

Our study has shown that being directly admitted to the ICU from the ED rather than being transferred from regular wards, developing AKI, sepsis, MOF, and ARDS, or having an uncontrolled malignancy are all predictive factors for short-term mortality in critically ill cancer patients nonelectively admitted to the ICU. Vasopressor use and mechanical ventilation were also predictors of mortality.

## 1. Introduction

The number of patients with malignancies has been increasing steadily throughout the past years. As a matter of fact, according to the World Health Organization (WHO), there has been 18.1 million new cancer cases and 9.6 million cancer-related deaths, with a prediction to reach 29.4 million new cancer cases in 2040 [1]. These data are according to the most recent WHO report in 2018. With the recent advances in the screening, diagnosis, and treatment of cancer, there has been a worldwide decrease in mortality rate among this patient population and the overall survival has been improving significantly [2]. In fact, the growing number of cancer patients alive means an increase in the probability of their need for critical care [3]. There has been a steady increase in the number of oncology patients admitted to the intensive care units (ICUs), either electively (e.g., after surgery) or nonelectively (i.e., for life-threatening complications) [2].

The general opinion is that intensive and critical care for cancer patients is futile, with the majority of patients not surviving and placing a burden on the ICUs, as well as the patients and their families [3]. Patients diagnosed with advanced cancer were not previously allowed to be admitted to the ICU, due to their dismal survival rates [4]. In fact, studies by the Society of Critical Care Medicine have led to guidelines that suggested that patients with metastatic cancer or who are unresponsive to chemotherapy or radiotherapy were not eligible for admission. The guidelines recommended limited care for patients with metastatic cancer admitted for certain complications such as infections or respiratory failure [5]. In addition, studies have shown that having metastatic cancer was the most important patient-related factor that led to ICU admission refusal [6]. However, studies have shown that an increasing number of cancer patients are surviving their ICU stay and are living more or less normal lives. These are mainly related to the improved diagnostic tools, the proper screening of patients requiring intensive care, and the decrease in cancer-related mortality [7].

Thus, it is important to study and evaluate the factors associated with both short- and long-term mortality in critically ill cancer patients nonelectively admitted to the ICU. This would help us further understand critical care in cancer patients as well as aid in the decision to admit a cancer patient into the ICU. Eventually, the ultimate goal is to be able to develop admission criteria for this study population, which would guide intensivists and oncologists in their decision-making capabilities. In this manuscript, we intend to describe demographic, clinical, and survival data and to identify factors associated with short- and long-term mortality in critically ill advanced cancer patients nonelectively admitted to medical ICUs.

## 2. Materials and Methods

This was a prospective single-institutional study involving critically ill cancer patients nonelectively admitted to the intensive care units (Medical Intensive Care Unit, Respiratory Care Unit, and Neurological Intensive Care Unit) at the American University of Beirut Medical Center (AUBMC), a tertiary cancer center, receiving patients from the Middle East and North Africa (MENA) region. AUBMC is a private, not-for-profit, teaching center of the university's Faculty of Medicine. It includes a 420-bed hospital with 25 beds in the intensive care units. According to the hospital registry, an average of 280 patients are admitted to the ICU every year. The study was strictly observational, and every clinical decision was left at the discretion of the intensivist and attending physician. The study was conducted according to the ethical principles stated in the Declaration of Helsinki (2013). IRB (Institutional Review Board) at the American University of Beirut reviewed the study proposal, and IRB approval was granted prior to data collection. Informed consent was taken, and data collected were kept confidential and no patient identifiers were used throughout the study.

Recruitment of eligible patients began in August 2015 and was completed in March 2019. Patients were followed up from day 1 of ICU admission until 30 days after discharge from the ICU or until death, whichever occurred first. Conditions at ICU discharge and at 30 days after discharge from ICU were the main outcomes of interest.

The principal investigator and his research associates screened all new admissions to the ICU on a daily basis to identify eligible patients for the study. All adult patients (≥18 years old) with a definitive diagnosis of hematological or solid malignancy, who required nonelective admission to the intensive care units (ICUs) at AUBMC, were evaluated. Cancer patients electively admitted to ICU for monitoring following a surgical procedure were excluded from the study. Patients who have been in complete cancer remission for more than 5 years were also excluded.

Demographic, clinical, and laboratory data including age, sex, hospital location before ICU admission, main reasons for ICU admission, and the need for ventilator support or inotropes usage were recorded. Comorbidities and cancer- and treatment-related information were all collected from the charts.

## 3. Results

### 3.1. Characteristics of the Study Population

Two hundred seventy-two cancer patients were enrolled in the study between August 2015 and March 2019. The median age of the study cohort was 65 years along a range of 18–92, with 67.3% of the population being males. In terms of the type of malignancy, 68.8% of the patients had a solid malignancy, compared to 31.3% with a hematological malignancy. In terms of malignancy status, controlled malignancy was defined as patients in partial or complete remission, with or without maintenance treatment, and uncontrolled malignancy as patients in progression, receiving any treatment modality (chemotherapy, immunotherapy, radiation therapy, or combination). 26.1% of patients had an uncontrolled malignancy, compared to 73.9% with a controlled malignancy. The major reasons for unplanned ICU admission were sepsis/septic shock (54%). Code status consisted of a high rate of 72.1% of patients with full code and 27.9% with a Do-Not-Resuscitate/Do-Not-Intubate (DNR/DNI) code status. This notable number of patients with DNR/DNI code is due to the culture and family beliefs, limiting the admission under the palliative care team in these cases and requiring full medical care. 160 patients were undergoing curative treatment (60.6%), compared to 104 (39.4%) receiving palliative treatment. Patients were considered as either a curative or palliative admission depending on the physicians' notes. Of all the patients, 66.2% were admitted directly from the emergency department (ED) and 33.8% were transferred from regular wards into the ICU. In terms of treatment, 60.8% of patients did not receive chemotherapy within 30 days prior to admission, compared to 39.2% who did. Only 9 patients of the 78 who received radiotherapy received it within 30 days prior to ICU admission, and one hundred three patients (38.9%) underwent surgery (Table 1). Patients having disease progression were considered as uncontrolled disease group.

### 3.2. Outcomes

**Figure 1:**
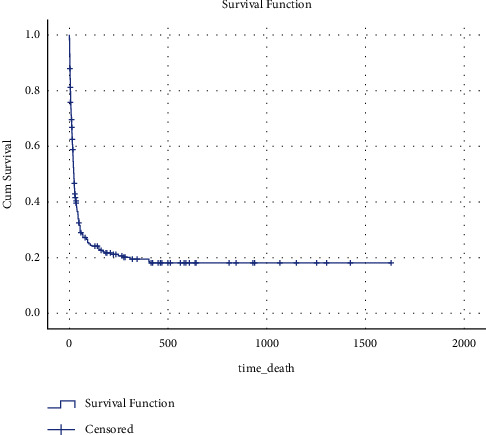
Overall survival.

The mean length of stay in the ICU was 14 days, with the median being 7 days (IQR 1–153). Mortality in ICU was 43.4%, and 25.4% of deaths occurred within one month of ICU discharge, totaling up to 67.6% mortality from the day of admission to one-month after discharge. The median overall survival (OS) was 22 days since the date of ICU admission, with a 3-month OS of 26.4% and 6-month OS of 21.7% (Figure 1). Table 1 depicts the complications along with the management of care in the ICU.

### 3.3. Univariate Analysis

Univariate comparisons of the clinical characteristics and outcomes of patients were performed. Reason for ICU admission, timing of admission, chemotherapy or radiotherapy within 30 days of ICU admission, anemia, leukopenia, leukocytosis, thrombocytopenia, and creatinine level prior to admission, code status, and curative versus palliative therapy were not found to be significant predictors of mortality in the study population.

Development of sepsis, AKI, MOF, or ARDS (*p* < 0.05) during their ICU stay was associated with increased mortality at discharge from the intensive care facility, with mortality rates of 23.9, 15.8, 10.6, and 15.8%, respectively (Table 2). Patients with uncontrolled malignancy status had worse outcomes, with a mortality rate of 58.6% at discharge from the ICU, compared to patients with controlled tumors (37.4%) (*p*=0.029). Direct admission from the ED was associated with a higher risk of mortality (48.9%) than being transferred from the floor (32.6%) (*p*=0.014).

Additionally, mortality in patients with solid malignancies (47.6%) was higher than those with hematologic malignancies (34.1%) (*p*=0.0048). Mortality was the highest in lymphoma (43.2%) and lung cancer (41.3%) patients, followed by leukemia patients (23.8%) (*p*=0.029).

### 3.4. Multivariate Analysis

Multivariate analysis identified six predictors of mortality in the study population. Sepsis (HR, 5.05, 95% CI, 1.633–15.652, *p*=0.05) during the ICU stay was associated with the highest risk of mortality, while vasopressor use (HR, 2.144; 95% CI, 0.971–4.733; *p*=0.05) was the lowest (Table 3).

## 4. Discussion

This study provides interesting data regarding the status of critically ill cancer patients in Lebanon, which is possibly a representative of the MENA region. It is important to determine the similarity between our cohort and cohorts from other studies, in order to subjectively compare the different populations.

Pooling together patients with solid malignancies and hematological malignancies was always done in previous studies of the MENA regions. As a starting point, these percentages should be close to be able to compare cohorts. Other studies have described different ranges in proportion of solid versus hematological malignancies (from 64 to 93% of solid tumors and 7 to 26% of hematological malignancies) [3, 4, 8, 9].

Lung cancer and leukemia were the most common malignancies in our study population (16.9 and 15.4%, respectively). The notable number of lung cancer admissions to the ICU is a reflection of the high morbidity and mortality of lung cancer, as it is the leading cancer type causing death [10, 11]. While some studies reported leukemia and non-Hodgkin's lymphoma as the most common malignancies, other studies reported malignancies such as non-Hodgkin's lymphoma and gastrointestinal tumors [3, 8, 9]. In studies targeting specifically solid tumors, lung cancer was the most common malignancy in some, while gastrointestinal, colorectal, and breast cancer were mentioned in a few studies as well [2, 12]. This further strengthens the reliability of our data, considering that our cohort is similar to previous studies.

The major reasons for admission into the ICU in our study were sepsis/septic shock and respiratory failure. This finding has been uniform across all studies, with sepsis/septic shock being the most common reason for admission in most studies. Auclin et al., Aygencel et al., Faucher et al., and many others have reported the same results [2, 3, 13]. In patients with lung cancer, some studies have shown pneumonia and respiratory failure to be the most common reason for ICU admission [14]. Heo et al. reported respiratory failure and neurologic deterioration as the most common causes of ICU admission [15].

Our study looked into patients who have received chemotherapy within 30 days prior to admission. In our study, 39.2% of patients (*n* = 78) received chemotherapy recently before being admitted to the ICU. Chang et al. reported a similar number in 2014, with 40% of cancer patients receiving chemotherapy during that period [14]. Heo et al., on the other hand, reported a 75% rate of active treatment within the last 30 days in 2015, with a patient population of 116 [15]. Other studies have mostly collected data on the number of patients who have undergone, or are undergoing, chemotherapy treatment, regardless of when the last chemotherapy dose was. They mostly reported the total percentage of patients who have received chemotherapy, and these include a large range between 55 and 79% [2–4, 16, 17].

Our study reported an ICU mortality rate of 43.4%, with the number rising to 59% within 30 days of ICU discharge. The study also reported an overall survival of 22 days since the day of ICU admission. Aygencel et al. reported a mortality rate of 55% in 2014, Anisoglou et al. reported an ICU mortality rate of 47.4% in their study population in 2013, and Oeyen et al. reported a rate of 38% in 2013, to name a few studies [3, 8, 16]. In fact, Auclin et al. reported a wide range of ICU mortality among studies, ranging between 24 and 75% [2]. Our data fall right in the middle of the reported range and comply with previous studies regarding this patient population. The mean length of stay in our ICU was 14 days with an interquartile range of 1 to 120 days. Other studies have reported different mean lengths of stay in the ICU, with a mean range between 4 and 10.8 days [3, 9, 18–22].

Conversely, we had an increased number in patients with a code status of DNR/DNI admitted to the ICUs. This is due to the beliefs that stopping all medical treatments in terminal-stage cancer patients is against religion and culture in our society. For this reason, family members go to full medical care, without resuscitation nor intubation, hence the need to introduce palliative care early. Their main goal is to discuss the goals of care with the patient and explain the overall situation and risks to avoid futile care and stress to family members taking decisions.

Hawari et al. ran the multivariate analysis to identify factors that led to the ICU admission, as well as factors that led to a poor outcome. When looking into factors that affect the likelihood of ICU admission, they found that having a hematological malignancy, receiving recent chemotherapy, advanced cancer stage, and smoking to be strong predictors of ICU admission [4]. In fact, it has been shown in many studies that having a hematological malignancy increases chances of complications, more so than solid tumors. Also, receiving chemotherapy predisposes the patients to cytopenia, which increases the chances of infection and sepsis, indirectly leading to increased mortality. Chemotherapy treatment within 30 days prior to admission was not shown as a predictor of outcome in both univariate and multivariate analyses, and it was not explicitly implicated in poor outcome in previous studies with similar cohorts. This could be explained by the management of febrile neutropenia cases with GCSF injections prophylactically and subsequently sparing ICU admissions.

A study by Faucher et al. looked into the outcomes of patients with hematological malignancies admitted to the ICU, and their multivariate analysis found invasive mechanical ventilation and renal replacement therapy for allogenic hematopoietic stem cell transplant patients, performance status and mechanical ventilation for neutropenic patients, and renal replacement therapy for patients receiving mechanical ventilation to be all the factors associated with a poor short-term outcome [13]. Gupta et al. found SOFA scores, hypotension, and septic shock to be predictors of mortality in their multivariate analysis [17]. Sepsis, acute respiratory failure, high doses of catecholamines, renal replacement therapy, and high SAPS II scores were found to be predictive of mortality in multivariate analysis done by Horster et al. in 2012 [23].

Aygencel et al. have also found the severity of the clinical illness to be predictive of mortality in multivariate analysis in their population. They estimated the severity of the illness using the APACHE II score [3]. In our study, the status of the malignancy was found to be predictive of mortality, with an uncontrolled malignancy predisposing to poorer outcomes. This was also shared by Heo et al., who found that having an uncontrolled malignancy status is a predictor of mortality in their study population [15].

The presence of multiorgan failure as a predictor of mortality has been shown and reported by numerous studies. Hwang et al. found that among patients with lung cancer, development of multiorgan failure was an independent factor associated with mortality [24] Parakh et al. also found that the having multiorgan failure and the number of organs that have failed are predictors of mortality in patients with any malignancy [22]. This was also shown by Soares et al. and is consistent with the findings in our study population [9]. Hence, it seems that many studies support the notion that developing multiorgan failure, whether in the ICU or prior to admission, is a key factor in cancer patients admitted to the ICU.

Finally, our results showed that being directly admitted from the emergency department (ED) was associated with a higher mortality rate. Previous studies have shown that the duration a patient spends outside of the ICU before being transferred to it is associated with higher mortality. The data showing that late ICU admission from the regular wards is associated with a higher mortality are shared by many studies, including those of Aygencel et al. and Soares et al. [3,9]. Some other studies have found a lengthy stay before ICU admission to be predictive of mortality in univariate analyses, but not in multivariate analyses. A possible explanation of the results of our study could be related to the more critical nature of patients presenting to the ED in our institution and to the optimal timing during which patients are transferred from regular wards to the ICU. Besides, it could be explained by the delay of presentation to the ED due to problems in the healthcare system of our lower-middle-income country, Lebanon, based on the National Social Security Fund (NSSF) and private insurance companies that do not cover ED medical care fees. Hence, patients try to avoid admissions through the ED, try ambulatory treatment with oral antibiotics in case of febrile neutropenias or sepsis, and present later to the ED with an advanced sepsis requiring ventilatory and hemodynamic support.

Nonetheless, our study results must be interpreted with caution and a number of limitations should be borne in mind. First, the study was limited to 30-day mortality that remains a short-term outcome. Second, as some illness scores, like APACHE and SOFA, were not used upon admission to ICU, having some objective data was not possible. Finally, heterogeneity of various cancers made interpretation of mixed results difficult.

## 5. Conclusion

Our study has shown that being directly admitted to the ICU from the ED, rather than being transferred from regular wards, developing AKI, sepsis, MOF, and ARDS, or having an uncontrolled malignancy are all predictive factors for short-term mortality in critically ill cancer patients nonelectively admitted to the ICU. Vasopressor use and mechanical ventilation were also predictors of mortality. While part of our results was in compliance with other studies, others provided additional information to investigate more. Interestingly, our study has shown that direct admission from the ED is a negative prognostic factor, which has not been reported before.

We believe it is important to continue following up with patients in different institutions and to compare variables and obtain other information such as patients' APACHE II score or sarcopenia malignancy. Finally, we believe there is a critical need for identifying predictive factors for ICU admissions of this population, as its importance in avoiding futile care and better management of these cases in integrating palliative care earlier is needed. These ICU admission criteria can serve as guidelines for admission and can help the physician in making an optimal decision in the patient's care.

## Figures and Tables

**Table 1:** Patient characteristics and ICU stay.

Patient characteristics	
Number of patients, *N*	272

Age, yr.	
Mean	64.25

Type of malignancy, *n* (%)	
Lung cancer	46 (16.9%)
Leukemia	42 (15.4%)
Lymphoma	37 (13.6%)
Others	147 (54.1%)

Stage, *n* (%)	
Solid and hematologic tumors other than leukemia	
Low-intermediate (stages I–III)	49 (22.8%)
High (stage IV)	166 (77.2%)
Leukemia^*∗*^	
Low or intermediate risk	12 (30.8%)
High risk	27 (69.2%)

Malignancy status, *n* (%)	
Controlled	198 (73.9%)
Uncontrolled	70 (26.1%)

Aim of treatment, *n* (%)	
Curative	160 (60.6%)
Palliative	104 (39.4%)

Reason for ICU admission, *n* (%)	
Sepsis	147 (54%)
Respiratory failure	90 (33.1%)
Altered general status	20 (74%)
Hemorrhagic shock	15 (5.5%)

Mode of admission, *n* (%)	
Through ED	180 (66.2%)
Transfer from floor	92 (33.8%)

Laboratory values, *n* (%)	
Anemia	226 (83.1%)
Leukopenia	58 (21.3%)
Thrombocytopenia	107 (39.3%)
Renal impairment	103 (38.1%)

ICU complications, *n* (%)	
Sepsis	226 (83.1%)
Invasive fungal infection	20 (7.4%)
ARDS	63 (23.2%)
AKI	152 (55.9%)
MOF	94 (34.7%)

ICU care, *n* (%)	
Mechanical ventilation	157 (57.7%)
Vasopressor use	185 (68%)
Antibiotic use	266 (97.8%)
Dialysis	45 (16.5%)

ICU length of stay, day	
Mean	14
Median (range)	7 (1–153)

^*∗*^According to Rai classification.

**Table 2:** Univariate analysis of ICU outcome.

Variable	Mortality rate (%)	*p* value
Sepsis	23.9	0.046
AKI	15.8	<0.005
ARDS	14.3	0.014
Multiorgan failure	10.6	<0.005
Uncontrolled malignancy	37.4	0.029
Direct admission from ED	48.9	0.014
Solid malignancy	47.6	<0.005

**Table 3:** Multivariate analysis of ICU outcome.

Cox regression variable	HR	95% CI	*p* value
Sepsis	5.05	1.63–15.65	0.005
Uncontrolled malignancy	3.18	1.33–7.61	0.009
ARDS	2.62	1.02–6.68	0.044
Multiorgan failure	4.85	1.99–11.93	0.001
Use of vasopressors	2.14	0.97–4.73	0.05
Use of mechanical ventilation	2.873	1.352–6.104	0.006

## Data Availability

The data that support the findings of this study are available from the corresponding author upon reasonable request.
